# The Cardiometabolic Multimorbidity Risk Profile of Females Living in Glendale, ILembe District of Kwa-Zulu Natal

**DOI:** 10.3390/ijerph21111436

**Published:** 2024-10-29

**Authors:** Bridget Grobler, Terry Jeremy Ellapen

**Affiliations:** Department of Sport, Rehabilitation & Dental Science, Pretoria West Campus, Tshwane University of Technology, Pretoria 0001, South Africa; ellapentj@tut.ac.za

**Keywords:** cardiometabolic multimorbidity, overweight, blood pressure

## Abstract

Background: Empirical studies have indicated that cardiometabolic multimorbidity risk profiles are upsurging among the South African population. However, there is a limited number of studies that have been conducted to validate these findings in rural communities. Aim: To study the prevalence of cardiometabolic risk factors of females residing in rural Glendale in the ILembe District, South Africa. Methods: One hundred females (aged 18–40 years) voluntarily participated in a cross-sectional observational study. All participants completed an ISAK somatotype profiling, and measured their fasting cholesterol, glucose, blood pressure and resting heart rate. Results: Participants’ average age, body mass index, stature, and body mass, were 28.34 ± 7.07 years, 29.5 ± 8.06 kg/m^2^, 157.26 ± 6.09 cm, and 72.9 ± 21.12 kg, respectively. The cohort had a mean waist circumference of 94.2 ± 22.1 cm, hip circumference of 108.4 ± 20.9 cm, and waist-to-hip ratio of 0.86. Participants’ mean heart rate, systolic blood pressure, and diastolic blood pressure were 86.31 ± 8.68 bpm, 116.79 ± 16.34 mmHg, and 82.14 ± 10.87 mmHg, respectively. Eleven participants recorded a resting heart rate greater than 100 bpm. Average blood glucose, total cholesterol, low-density lipoprotein, and high-density lipoprotein recorded were 4.87 ± 1.26 mmol/L, 3.78 ± 0.94 mmol/L, 1.76 ± 1.86 mmol/L, and 1.31 ± 0.4 mmol/L. Eighty-three participants HDL-C were below the recommended normative value of 1.55 mmol/L. Conclusion: The average participant presented as overweight, with elevated diastolic blood pressure, and a resting heart rate that has been proven to increase one’s cardiometabolic multimorbidity risk profile. Additionally, a small portion of the cohort were identified to be prediabetic and diabetic. Large proportion of participants had low HDL-C levels suggestive of poor cardiovascular disease protection.

## 1. Introduction

Cardiometabolic risks have been described as a collection of metabolic and cardiovascular abnormalities, which include abdominal obesity, insulin resistance, hypertension, dyslipidemia, and atherosclerosis [1]. This collection of abnormalities predisposes the person to cardiovascular diseases and non-insulin diabetes (diabetes mellitus type 2) [1]. Cardiovascular diseases are the primary cause of mortality worldwide [2]. In 2019, 32% of all global mortality (approximately 18 million people) was attributed to cardiovascular deaths [2]. The most alarming fact is that 75% of these cardiovascular deaths occurred in low- and middle-income countries, such as South Africa [2]. Cardiovascular diseases have been cited for 17.3% of deaths among South Africans [3].

Hypertension is the primary risk factor of cardiovascular deaths, associated with 13% of premature deaths globally [4,5]. In South Africa, hypertension is the primary risk factor for strokes (50%) and cardiac arrest (42%) [4]. Approximately 65% of South African females and 31% of males were identified to be overweight [5]. Tragically, 40% of South African females and 12% of males have been classified as obese [5]. Dyslipidemia is described as an increase in triglycerides, plasma cholesterol levels, or both, in combination with a low high-density lipoprotein cholesterol (HDL-C) level that influences the development of atherosclerosis [1]. Shisana and colleagues (2013) reported that 25% of the adult South African population have elevated plasma cholesterol levels and low-density lipoprotein cholesterol (LDL-C) levels in combination with low HDL-C levels and have been identified as having dyslipidemia [5]. In South Africa, approximately 4.2% of the population were aware they had high LDL-C, while many medical doctors and health scientists are of the opinion that South Africans are unaware of the poor cardiometabolic lipid profile status associated with cardiovascular diseases [5]. The prevalence of diabetes mellitus in South Africa has quickly increased from 4.5% in 2010 to 12.7% in 2019 [6]. Of the 4.6 million South Africans aged 20–79 years who are estimated to have had diabetes mellitus in 2019, 52.4% were undiagnosed [6]. Over a prolonged period of time, diabetes mellitus damages blood vessels in the eyes (leading to diabetic retinopathy), heart, kidneys (leading to diabetic nephropathy), and nerves (leading to diabetic neuropathy) [1].

Sewpaul and colleagues refer to a special clinical scenario, cardiometabolic multimorbidity, which describes the presence of two or more cardiometabolic risk factors in the same person [7]. Cardiometabolic multimorbidity increases the risk of mortality multiplicatively, where each additional disease doubles the risk [8]. Van Heerden and colleagues reported that many South Africans residing in the rural communities of Kwa-Zulu Natal have cardiometabolic multimorbidity [9]. However, there are limited empirical studies that identify the cardiometabolic risk among residents of rural communities in South Africa. This research aimed to study the prevalence of cardiometabolic risk factors among rural females residing in the Glendale area of the ILembe District of Kwa-Zulu Natal, South Africa, to aid the development of strategies for the prevention and reduction of cardiometabolic risk. Such strategies include awareness campaigns promoting foods that are less refined, early identification of cardiometabolic risks, and regular physical activity.

## 2. Materials and Methods

### 2.1. Method

The study adopted a cross-sectional observational design. One hundred participants aged between 18 and 40 years voluntarily participated in the study. The study was conducted in the rural Glendale area of the ILembe District in KwaZulu-Natal, South Africa. There were approximately 140 females within the aforementioned age strata residing in the Glendale area, but only 100 females volunteered to be involved in the study, which equates to 71.4%. According to Terre-Blanche and colleagues, the statistical rule of thumb regarding the lowest percentage of participants volunteering in an empirical study yielding a strong power of significance of the findings is 30%, which this sample size exceeded [10]. Many residents (both adolescent and adult males and females) relocate to urban communities in search of employment opportunities and a better life. Glendale community is a rural mountainous landscape approximately 100 km away from the closest urban town (eThekwini). The local medical facility is a clinic primarily focusing on pre- and post-natal care and attending to musculoskeletal injuries incurred from agriculture. Agriculture is the primary means of income for this rural community. Anecdotal reports indicate that cardiometabolic screening and risk stratification does not occur often in this community. The aim of this research study is to determine the prevalence of cardiometabolic risk factors among female residents of the Glendale area of the ILembe District of Kwa-Zulu Natal. The findings of this study will be employed to develop strategies to prevent and reduce the cardiometabolic risk factors in this community. Prior to data collection, gatekeeper permission from the ILembe Royal Court and informed consent from individual participant were secured. The study received ethical approval from the Tshwane University of Technology, Pretoria, South Africa (REC04/9.2.1).

### 2.2. Participants

Participants were females whose responsibilities included chaperoning infants and the elderly, cleaning their homes, washing clothing, collecting firewood from the surrounding forest, retrieving water from the river, cooking, and farming. The residents of the Glendale area are fluent in English speaking, reading, and writing. However, their health education and knowledge of cardiometabolic risks and diseases are limited. The limited finances of the Glendale residents make regular health screening and cardiometabolic risk stratification impossible. The primary aim of residents of the ILembe district is survival. Kurten et al. has stated that the ILembe District has been identified as an impoverished district of South Africa [11].

### 2.3. Data Collection Protocols and Materials

Kinanthropometry is the study of the individual’s shape, size, proportionality, and body composition to better comprehend the process of growth, the effect of physical activity, and nutrition [12]. The value of gathering kinanthropometric data provides us with knowledge of the cohort’s body mass index (BMI), percent body fat, and percent fat-free mass. Body mass index and percent body fat are commonly used to classify whether individuals are underweight, normal weight, overweight, and various grades of obesity [12,13]. The calculation of BMI and percent body fat will be used to determine whether this cohort has these cardiometabolic risk factors.

Kinanthropometrical assessments were conducted according to the standard protocols of the International Society for the Advancement of Kinanthropometry (ISAK) [12]. These kinanthropometrical measurements included stature, body mass, skinfolds, breadths, and girths. Stature was measured using a portable stadiometer (Holtain Limited, Crosswell, UK). The participant stood upright with their head held in the Frankfurt plane. Stature was measured to the nearest 1 mm. Body mass was measured in kilograms to the nearest 0.1 kg using a portable electronic scale (Ps07 Electronic Scale, Beurer, Ulm, Germany). Participants wore their normal clothing without shoes when their body mass was measured. Anatomical skinfold sites included the triceps, biceps, subscapular, supraspinal, iliac crest, abdominal, anterior thigh, and medial calf, which were measured with a Harpenden skinfold calliper (Holtain Limited) to the nearest 0.1 mm with a constant pressure of 10 g/mm^2^. The breadth measurements included the humerus, wrist, femur, and ankle and were taken with a Holtain bicondylar calliper (Holtain Limited) to the nearest 0.1 mm. Girth measurements recorded included the relaxed arm, flexed arm, chest, abdomen, waist, gluteus, and mid-thigh and were taken with a Lufkin metal tape (Cooper Industries, Cleveland, OH, USA) to the nearest 0.1 cm. All measurements were taken by the same two Level 2 ISAK-certified anthropometrists twice on the right-hand side of the body. This was to ensure high inter-rater reliability. The average values of these measurements were used in the statistical analysis. Body fat percentage and muscle mass percentage were calculated by the established formula of ISAK. Further, participants’ resting heart rate, blood pressure, fasting blood glucose, and cholesterol tests using the standard protocols of the Accu-Check Instant (Accu-Check, Roche Diabetes Ltd., Geneva, Switzerland) were assessed [14]. Resting heart rate and resting blood pressure were measured using an electronic blood pressure monitor.

### 2.4. Statistical Analysis

All data were analyzed using the Statistical Package for Social Sciences (SPSS) version 27.0 for Windows (SPSS Inc., Chicago, IL, USA). Descriptive statistics were used to determine the cardiometabolic risk profile of the participants. Descriptive interrogation involved means, standard deviation, and percentage fluctuations. Inferential statistical included *t*-test analyses. Probability was set at *p* ≤ 0.05.

## 3. Results

The results will describe the cohort’s demographic characteristics (Table 1), clinical measures (Table 2), and clinical measures stratified via mean arterial pressure (Table 3).

### 3.1. Demographic Characteristics of Participants

Participants’ average age, body mass index (BMI), stature, and body mass were 28.34 ± 7.07 years, 29.5 ± 8.06 kg/m^2^, 157.26 ± 6.09 cm, and 72.9 ± 21.12 kg, respectively (Table 1). Participants’ age ranged from 18 to 40 years, body mass between 45.2 and 135.9 kg, and height between 147.5 and 176 cm (Table 1). The cohort had a mean waist circumference of 94.2 ± 22.1 cm, hip circumference of 108.4 ± 20.9 cm, and waist-to-hip ratio of 0.86. There were 29 participants whose waist circumference exceeded 100 cm, which fell short of the expected frequency (X^2^ = 0.001). Nine participants’ percent body fat exceeded 30%, indicative of overweight and obesity status. These nine participants fell short of the expected frequency (X^2^ = 0.001).

**Table 1:** Participants demographic characteristics (n = 100).

Demographic Variables	Measurements
Age (years)	28.34 ± 7.07
Body mass (kg)	72.99 ± 21.12
Stature (m)	1.57 ± 0.06
Body mass index of mother (kg/m^2^)	29.5 ± 8.06
Percent fat mass (%)	31.4 ± 10.7
Fat free mass (%)	68.57 ± 10.7

### 3.2. Cardiometabolic Risk Profile (n = 100)

The average heart rate of 86.31 ± 8.68 bpm (Table 2) falls within normal ranges (60–100 bpm) according to the standards as set out by the American Heart Association [13]. Tachycardia (a heart rate greater than 100 bpm) was present in 11 out of the 100 participants (X^2^ = 0.01). Normative resting diastolic blood pressure (DBP) ranges between 70 and 79 mmHg and systolic blood pressure (SBP) ranges between 120 and 129 mmHg, which is in line with the European Society of Cardiology’s norms [15]. Hypertension has also been defined as SBP > 140 mmHg and DBP > 90 mmHg, whereas elevated blood pressure has been introduced in 2024 as SBP between 120 and 139 mmHg and DBP between 70 and 89 mmHg [14]. The cohort’s mean value (82.14 ± 10.87 mmHg) exceeded the 80 mmHg threshold categorizing the population’s DBP as elevated blood pressure. The systolic blood pressure (SBP) value (116.79 ± 16.34 mmHg) indicated a normal SBP [15].

Average blood glucose levels were found at 4.87 ± 1.26 mmol/L, which can be classified as normal for the applicable age range according to the standards as set out by the World Health Organization in 2021 [2]. Twelve participants exceed the normative value of 5.5 mmol/L. Of these 12 participants, five had their fasting blood glucose levels ranging between 5.6–6.9 mmol/L, indicating prediabetic status, while the other seven were classified as diabetic. Average total cholesterol (3.78 ± 0.94 mmol/L), LDL-C (1.76 ± 1.86 mmol/L), and HDL-C (1.31 ± 0.4 mmol/L) can be classified as optimal for the age range applicable [2]. Nine participants’ total cholesterol was above 5.17 mmol/L, which is considered a borderline risk of cardiovascular diseases [2]. Of these nine participants, three had total cholesterol above 6.18 mmol/L, suggestive of increased predisposition to cardiovascular diseases. Two participants recorded LDL-C greater than the normative value of 2.6 mmol/L, of which one exceeded 3.4 mmol/L, suggestive of increased predisposition to cardiovascular diseases. Eighty-three participants’ HDL-C levels were below the recommended normative value of 1.55 mmol/L, suggestive of poor cardiovascular disease protection. Seventeen of these eighty-three participants recorded HDL-C below 1.03 mmol/L, suggestive of an increased risk of cardiovascular diseases.

**Table 2:** Average findings of resting measurement parameters (n = 100).

Testing Parameter	Average Value
Heart rate (bpm)	86.31 ± 12.1
Systolic blood pressure (SBP) (mmHg)	116.79 ± 16.34
Diastolic blood pressure (DBP) (mmHg)	82.14 ± 10.87
Blood glucose level (mmol/L)	4.87 ± 1.26
Total cholesterol level (TC) (mmol/L)	3.78 ± 0.94
Low density lipoprotein cholesterol (LDL-C) (mmol/L)	1.76 ± 1.86
High density lipoprotein cholesterol (HDL-C) (mmHg)	1.31 ± 0.4

The mean arterial pressure (MAP) value represents the quality of blood flow throughout the body with a normal value at 100 mmHg [16]. Higher values can be found when systemic vascular resistance is higher (e.g., conditions such as hypertension) or when resting heart rate is increased or stroke volume is decreased (common during heart failure, coronary artery disease, and pulmonary embolismic conditions) [16]. This value is calculated by adding one-third of the pulse pressure (pulse pressure = systolic pressure—diastolic pressure) to the amount of the diastolic blood pressure [16]. Table 3 displays the stratification of participants age, blood pressure, LDL-C, total cholesterol, and HDL-C based on their mean arterial pressure scores.

Table 3 displays the comparative differences between participants with normal MAP (n = 78) versus those with above-normal MAP (n = 22). The comparative analyses were completed using an independent *t*-test with the probability set at 0.05. Of the 100 participants, 22 displayed a MAP value of higher than 100 mmHg (Table 3) (*p* ≤ 0.0001). These 22 participants were older (31.04 ± 6.14 years) than the other 78 participants (27.62 ± 7.16 years) (*p* = 0.03) (Table 3). Furthermore, their (n = 22) average body mass (78.01 ± 21.21 kg) was heavier in comparison with the other 78 participants (71.65 ± 21.03 kg; *p* = 0.23), and their percentage body fat was lower (15.78 ± 7.16% vs. 19.84 ± 8.29%; *p* = 0.03) (Table 3). The participants who had MAP above normal levels recorded significantly higher systolic and diastolic blood pressure (*p* < 0.01). However, their total cholesterol, LDL-C, HDL-C, and non-HDL-C levels did not significantly differ from the group with normal MAP (*p* > 0.05) (Table 3).

**Table 3:** Comparison of the cohort blood pressure stratified against mean arterial pressure (n = 100).

Variables	Above Normal MAP (100 mmHg) (n = 22)	Normal MAP (60–100 mmHG) (n = 78)	*p*-Value
MAP (mmHg)	109.57 ± 12.49	89.46 ± 7.05	0.0001 *
SBP (mmHg)	137.76 ± 20.14	111.21 ± 9.21	0.0001 *
DBP (mmHg)	95.47 ± 9.18	78.59 ± 8.22	0.0001 *
Heart rate (bpm)	85.66 ± 10.57	86.48 ± 12.52	0.76
Age (years)	31.04 ± 6.14	27.62 ± 7.16	0.03 *
Body mass (kg)	78.01 ± 21.21	71.65 ± 21.03	0.23
Percent body fat (%)	15.78 ± 7.16	19.84 ± 8.29	0.03 *
Cholesterol (total serum) (mmol/L)	4.00 ± 1.20	3.51 ± 0.8	0.08
LDL-cholesterol (mmol/L)	1.41 ± 0.34	1.85 ± 2.07	0.11
HDL-cholesterol (mmol/L)	1.46 ± 0.51	1.28 ± 0.40	0.16
Non-HDL cholesterol (mmmol/L)	2.54 ± 0.77	2.23 ± 0.70	0.11

* Denotes significance greater than *p* < 0.05.

## 4. Discussion

The average participant was classified as being overweight due to the high average BMI, WHR, and high average body fat percentage which corresponds with van Heerden et al. [9]. The American Heart Association (AHA) classifies an individual’s BMI greater than 25 kg/m^2^ and 30 kg/m^2^ as overweight and obese, respectively [13]. Overweight and obesity are associated with an increased risk of numerous cardiometabolic diseases, such as cardiovascular diseases, diabetes mellitus, and cancer [17]. High WHR suggests excessive abdominal fat, which increases a person’s risk of diabetes mellitus [18]. Excess body fat accumulation may impair insulin signaling through cell autonomous mechanisms, thereby weakening the insulin action, and increasing insulin resistance [19]. Insulin resistance leads to elevated blood glucose levels (hyperglycemia), which, over time, result in prediabetes and type 2 diabetes [18,19]. Excessive accumulation of fat (adipose tissue) in the myocardium causes morphological and physiological alterations. Furthermore, the increased adipose tissue stimulates the secretions of various hormones, which creates a pro-inflammatory and prothrombotic state. This pro-inflammatory and prothrombotic state predisposes the person to coronary heart diseases [17]. Excess body fat raises the risk for several types of cancers, such as colorectal, esophageal, kidney, post-menopausal breast, uterine, and pancreatic cancers [17]. Manafe et al. reported that there was an upsurge in obesity trends among South Africans due to cultural beliefs, poor dietary consumption, and physical inactivity [20]. Many black South Africans’ traditional cultural belief is that an obese woman is attractive despite the health consequences [11]. The participants were from a rural community which still adheres to these cultural beliefs [11]. Overweight and obese people are at higher risk of developing cardiometabolic diseases (manifested in the form of cardiac arrests and/or cerebrovascular events (strokes), non-insulin diabetes mellitus, osteoarthritis, and cancers (endometrial, breast, and colon) [21].

The average diastolic blood pressure value was classified as elevated [15]. Increased diastolic blood pressure values are indicative of underlying conditions such as aortic stenoses, heart failure, dyslipidemia, and heart valve failure [21]. High diastolic blood pressure values are common among overweight and obese individuals, as reported by Liguori et al., as is the scenario in this study [21]. Accompanying the high diastolic blood pressure was an elevated resting heart rate, which is suggestive that the heart is working harder to supply blood to the body. Elevated diastolic blood pressure escalates the probability of cardiovascular diseases and manifests through cardiac arrest and cerebrovascular events [22]. The present cohort had low HDL-C levels, which assists the removal of other types of cholesterol in the blood vessels, preventing the development of atheroma. These other types (especially LDL-C and VLDL-C) are transported to the liver, where they are further processed and eliminated from the body. Therefore, low HDL-C levels increase the risk of developing atheroma, which may elevate blood pressure. Atheroma reduces the internal blood vessel lumen, reducing blood flow. In order to maintain a normal blood flow, blood pressure increases (hypertension). High blood pressure (diastolic pressure) is one of the risks of metabolic syndrome and cardiovascular diseases leading to cardiometabolic diseases [22,23]. An elevated heart rate increases blood flow, and blood pressure, which promotes the disruption of an atheroma, which results in acute coronary disease [13].

No prior literature has been conducted to determine the influential factors of mean arterial pressure (MAP), heart rate, and blood pressure specific to the female rural South African population. Elevated MAP exceeding 100 mm Hg suggests that there is extreme pressure in the arteries, which may lead to blood vessel rupture, blood clots and/or damage to the heart muscle. Furthermore, unfortunate clinical conditions that can result from untreated MAP are strokes, cardiac arrests, and sudden cardiac death [13]. The elevated MAP among older females is a clinical concern. Increased MAP influences higher oxygen demand of the heart, ventricular remodelling, vascular injury, and end organ damage, precipitating cardiac arrest and cerebrovascular events [24]. Elevated blood pressure has been attributed to increased arterial stiffness (decreased arterial elasticity) due to age [25,26]. Augmented arterial stiffness begins at the age of 30 years [25,26]. An increased body mass was also seen in those with increased MAP due to increased pressure on the circulatory system. More soft tissue mass also justifies increased arterial pressure to increase oxygen saturation of the tissue [27].

## 5. Conclusions

The average participant presented as overweight, with elevated diastolic blood pressure, MAP, BMI, WHR, and a resting heart rate that has been proven to increase one’s cardiometabolic multimorbidity risk profile. A small portion of the cohort was identified to be prediabetic and diabetic. A large proportion of the participants had low HDL-C levels, suggestive of poor cardiovascular disease protection. Sewpaul and colleagues reported that individuals with two or more cardiometabolic risks should be considered as having cardiometabolic multimorbidity, which represents a serious clinical concern [8]. The aforementioned clinical evidence is concerning, which warrants females residing in the Glendale area of the ILembe District to consider lifestyle changes. The evidence of this paper should be used to develop preventative measures aimed at raising public awareness of cardiometabolic risks and diseases. Potential strategies for the prevention and reduction of cardiometabolic risk include awareness campaigns promoting food that are less refined, early identification of cardiometabolic risks, and regular physical activity.

## Data Availability

Data is unavailable to the public due to institutional policy.
